# Retraction Notice

**DOI:** 10.5713/ajas.14.0761RT

**Published:** 2024-10-23

Asian-Australas J Anim Sci 2015;28(3):420–7

https://doi.org/10.5713/ajas.14.0761

The Animal Bioscience (AB) ethical committee investigated possible plagiarism in the aforesaid publication and discovered that a portion of the data was basically identical to that in another article (Mol Cancer Ther 2006 Sep 5(9):2398–407). According to the AB Ethics Guidelines, the article (AJAS Vol 28 No 3, pages 420–427) shall be retracted as of November 1, 2024.

Animal Bioscience’s editorial board deeply apologizes to Molecular Cancer Therapeutics and all coauthors.

